# Rs4074134 Near BDNF Gene Is Associated with Type 2 Diabetes Mellitus in Chinese Han Population Independently of Body Mass Index

**DOI:** 10.1371/journal.pone.0056898

**Published:** 2013-02-19

**Authors:** Xueyao Han, Yingying Luo, Xiuying Zhang, Chao Lv, Xiuqin Sun, Xiaomei Zhang, Xianghai Zhou, Xiaoling Cai, Qian Ren, Linong Ji

**Affiliations:** Department of Endocrinology and Metabolism, Peking University People's Hospital, Peking University Diabetes Center, Beijing, China; University of Hong Kong, China

## Abstract

Obesity and family history are the most important predictors for type 2 diabetes mellitus(T2DM) in the Chinese Han population. However, it is not known whether the genetic loci related to obesity are associated with the risk of developing T2DM in this population. The present case-control study evaluated the associations between five genetic loci for obesity and the pathogenesis of T2DM. The study included 1117 Chinese Han patients with T2DM, 1629 patients with pre-diabetes (impaired fasting glucose and impaired glucose tolerance, IFG/IGT) and 1113 control subjects residing in Beijing. Five genetic loci including rs2815752 near NEGR1, rs10938397 near GNPDA2, rs4074134 near BDNF, rs17782313 near MC4R and rs1084753 near KCTD15 were genotyped. The results showed an association between rs4074134-BDNF minor allele and T2DM irrespective of age, gender and body mass index (BMI) (OR = 0.87; 95%CI: 0.77–0.99, P = 0.04). This SNP was also associated with pre-diabetes (OR = 0.87; 95%CI: 0.77–0.97, P = 0.01) independently of age, gender and BMI. No associations were found between diabetes or pre-diabetes and any of the other SNP loci studied. Genotype–phenotype association analysis (adjusting for age and gender) showed rs4074134-BDNF to be associated with BMI, waist circumference, fasting and postprandial plasma glucose, fasting serum insulin, and HOMA-IR in subjects without T2DM. However, fasting and postprandial plasma glucose were the only significant factors after adjusting for BMI. These results suggest that the common variation of BDNF (rs4074134) is associated with T2DM independently of obesity in Chinese Han population. This variant also has an effect on plasma glucose concentration, BMI and insulin sensitivity.

## Introduction

Type 2 diabetes mellitus (T2DM) is characterized by chronic hyperglycemia and defects in the secretion and/or actions of insulin. Genetic and environmental factors play important roles in the development of T2DM. Recent studies indicate that the prevalence of T2DM and obesity are dramatically increasing. It has been estimated that 92.4 million adults in China have diabetes, and that 148.2 million adults have pre-diabetes [1]. The same study identified a family history of T2DM and obesity as two of the most important risks for T2DM in Chinese patients [1].

The increasing prevalence of obesity is caused by the excessive calorie intake and diminished physical activity in the modern environment. However, available evidence also suggests a significant genetic contribution to adiposity [2]. The heritability for high body mass index (BMI) and large waist circumference is high (h^2^:0.60–0.63), and shared genetic factors have been identified in Chinese subjects patients that determine BMI, glucose homeostasis traits and insulin resistance index [3]. During the last 5 years, increasing numbers of genetic loci associated with obesity and/or BMI have been identified as a result of the genome-wide association study (GWAS). These loci include: *FTO*, *Mc4R*, *TMEM18*, *NEGR1*, *GNPDA2*, *BDNF*, *MTCH2* and *BCDIN3D–FAIM2* [4]–[11]. It is widely recognized that, *FTO*, which increases the risk for obesity, is also a susceptibility gene for T2DM [12]–[16]. It is notable that the association between *FTO* and T2DM remains statistically significant in Asian population even after adjusting for BMI [15],[17] but this is not the case in European studies [12],[13]. The difference may be attributed to the different genetic background among races.

Interventions, including life style modification and anti-diabetic drugs such as metformin, sulphonylurea and insulin, that have strong effects on BMI, would be expected to distort the relationship between BMI, genetic variation and T2DM. It is, therefore, possible that adjustment for BMI during logistic analysis in previous association studies identifying susceptibility genes for T2DM may have masked important risk loci for T2DM.

For these reasons it was of interest to evaluate the contribution of other loci associated with obesity in Chinese Han population. There is also a large population of subjects with pre-diabetes (impaired fasting glucose (IFG) or/and impaired glucose tolerance (IGT)) who are not currently taking anti-diabetic drugs. We, therefore, designed a case-control study consisting of controls with normal oral glucose test (OGTT) and patients with pre-diabetes in an attempt to identify associations between T2DM and five genetic loci near *NEGR1, GNPDA2, BDNF, MC4R and KCTD15* that have previously been reported to be associated with obesity or/and BMI [8]–[11].

## Materials and Methods

### Participants

A total of 3919 subjects of Northern Han Chinese ancestry residing in Beijing were included in the study. All subjects had attended the Endocrinology Departments of hospitals in Beijing. The population included 2806 unrelated subjects,1177 subjects with T2DM and 1629 with pre-diabetes. Diabetes and pre-diabetes were diagnosed in accordance with the 1999 World Health Organization criteria. Patients diagnosed with T2DM or pre-diabetes before 30 years of age, with a body mass index (BMI)>35 kg/m^2^, or clinical findings consistent with type 1 diabetes or other specific forms of diabetes (e.g., maturity onset diabetes of the young) were excluded from the study.

An additional 1113 control subjects >40 years of age, and with a BMI≤35 kg/m^2^ were recruited from Beijing. These subjects had normal glucose tolerance confirmed by a 75-g oral glucose tolerance test (OGTT) according to the 1999 World Health Organization criteria (fasting plasma glucose <6.1 mmol/L and 2-h plasma glucose <7.8 mmol/L), and had no family history of T2DM (3). The baseline clinical characteristics of subjects in all three groups are summarized in Table 1.

**Table 1:** Subject characteristics.

Characteristic	Controls(n = 1113)	Pre-diabetes(n = 1629)	T2DM(n = 1177)	P-value
M/F	316/797	689/940	504/673	<0.001
Age, years	53±10	52±10	60±10	<0.001
BMI, kg/m^2^	25.3±3.2	26.2±3.0	25.3±3.2	<0.001
Waist circumference, cm	84±9	89±9	87±9	<0.001
FPG, mmol/L	5.2±0.4	5.9±0.6	7.8±2.5	<0.001
Fasting serum insulin, mU/L	6.6 (4.8–9.4)	9.2 (6.3–13.4)	–	<0.001
HOMA-IR	1.5(1.1–2.2)	2.4(1.6–3.5)	–	<0.001
HOMA-β	82.6 (60.0–114.0)	79.5 (53.5–115.4)	–	<0.001

Data are shown as means ± standard deviation or median (interquartile range).

P-values represent significance differences between patients and controls.

The distribution of males/females was analyzed using Pearson's chi square test.

Quantitative variables were compared using ANOVA or Student's *t* test.

FPG: fasting plasma glucose; BMI: body mass index; HOMA-IR: homeostasis model assessment of insulin resistance; HOMA-β: homeostasis model assessment of β cell function.

All participants provided written informed consent, and the study protocol was approved by the Ethics Committee of Peking University People's Hospital.

### Anthropometric and biochemical measurements

All subjects were examined in the morning after a 10 to 12 h overnight fast. Height, weight, waist circumference, hip circumference and blood pressure were measured. Blood samples were collected to measure fasting plasma glucose, and fasting serum insulin. Plasma glucose levels were measured using the glucose oxidase-peroxidase method. Fasting serum insulin levels were measured using a radioimmunoassay. Using the homeostasis model assessment (HOMA), insulin resistance index (HOMA-IR) was calculated as fasting insulin (mU/L)×fasting plasma glucose (mmol/L)/22.5, and β cell function (HOMA-β) was calculated as fasting insulin×20/(fasting plasma glucose - 3.5) [18].

### DNA extraction and genotyping

Genomic DNA was extracted from peripheral blood leukocytes using a precipitation method. As rs2815752 near *NEGR1*, rs10938397 near *GNPDA2*, rs4074134 near *BDNF*, rs17782313 near *MC4R* and rs1084753 near *KCTD15* have been reported to be associated with obesity in Caucasian subjects [8]–[11]. We explored the roles of these SNPs in a Chinese population. SNP rs2815752 is located on chromosome 1 near (∼65 kb 5′-flanking of) *NEGR1*; SNP rs10938397 is in a gene desert ∼500 kb from *GNPDA2* on chromosome 4; SNP rs4074134 is located on chromosome 11 near (∼31 kb 3′-flanking of) *BDNF*; SNP rs17782313 is in 188 kb downstream of *MC4R*; SNP rs11084753 is located on chromosome 11 near (∼17 kb 3′-flanking of) *KCTD15*.

These SNPs were genotyped using a MassARRAY iPLEX system (MassARRAY Compact Analyzer, Sequenom, San Diego, CA, USA). Overall, 5% of the DNA samples were duplicated in genotyping for quality control. The call rates for all these SNPs were more than 95%,and the concordance rates were more than 98%.

### Statistical analysis

All statistical tests were performed using PLINK, version 1.07 (http://pngu.mgh.harvard.edu/~purcell/plink) and SPSS software version 16.0 for Windows (SPSS, Chicago, IL, USA. Continuous variables were presented as means and standard deviations (±SD) if normally distributed, or as medians (interquartile range) if non-normally distributed. Categorical variables were presented as numbers and percentages. Fasting serum insulin, 2-h serum insulin, HOMA-IR and HOMA-β were subjected to natural logarithm transformation to obtain a normal distribution prior to statistical analysis.

Between-group differences in clinical characteristics were analyzed using Student's *t* tests or ANOVA for continuous variables. Chi square tests were used to determine whether polymorphisms were in Hardy–Weinberg equilibrium. Differences in allele and genotype frequencies between the diabetic and control subjects were analyzed using Pearson's chi square test.

Logistic regression analysis was performed to calculate allele-specific odds ratios (OR) with 95% confidential intervals (CI) after adjustment for gender, age and BMI as covariates. Quantitative trait association analyses were performed using multiple linear regressions adjusting for age, gender and BMI as covariates. Association analyses of BMI and waist circumference were adjusted for age and gender. Because of the low frequency of minor alleles for homozygote of rs2815752 (less than cases and less than 10 controls) a dominant model was used in logistic and linear regression analyses.In all hypothesis tests, two-tailed values of *P*<0.05 were considered statistically significant.

### Power calculation

The power calculation was performed using Quanto software version 1.2.4 (University of Southern California, Los Angeles, CA, USA). Based on the prevalence of T2DM in China of 9.7% [1], and using a additive genetic model, this study had 80% power at *P*<0.05 to detect an effect size of 1.27 for rs2815752, 1.17 for rs10938397, 1.16 for rs4074134, 1.19 for rs17782313 and 1.18 for rs11084753 on T2DM.

## Results

### Association study on T2DM and pre-diabetes

The genotype frequencies of the five SNPs were in accordance with the Hardy–Weinberg equilibrium in the control groups except for rs10938397 (P = 0.04). Table 2 summarizes the results of the association study with T2DM. The minor allele frequencies (MAF) of these SNPs ranged from 0.1 to 0.44 in control subjects, which is comparable with values reported in the Hap Map database.

**Table 2:** Logistic regression analysis of association of five SNPs with type2 diabetes and pre-diabetes.

Chr	Genes	SNPs	MA	Control	T2DM			Pre-diabetes			Pre-diabetes and T2DM		
				MAF	MAF	OR (95%CI)	P-value	MAF	OR (95%CI)	P-value	MAF	OR (95%CI)	P-value
1	NEGR1	Rs2815752	G	0.10	0.09	0.95(0.76–1.17)	0.62	0.09	0.93(0.77–1.13)	0.48	0.09	0.94(0.78–1.11)	0.45
4	GNPD2	Rs10938397	G	0.32	0.32	1.02(0.90–1.17)	0.73	0.30	0.94(0.83–1.06)	0.29	0.31	0.96(0.86–1.07)	0.47
11	BDNF	Rs4074134	A	0.44	0.41	0.87(0.77–0.99)	0.04	0.40	0.87(0.77–0.97)	0.01	0.40	0.87(0.79–0.97)	0.009
18	MC4R	Rs17782313	G	0.24	0.23	0.82(0.82–1.09)	0.45	0.24	0.97(0.86–1.11)	0.68	0.24	0.97(0.86–1.07)	0.56
19	KCTD15	Rs11084753	G	0.35	0.37	1.11(0.97–1.27)	0.11	0.34	0.96(0.85–1.08)	0.48	0.35	1.01(0.91–1.11)	0.87

The additive model for minor allele was used and odds ratios were calculated by logistic regression analysis.

SNP: single nucleotide polymorphism; MAF: minor allele frequency; OR: odds ratio.

P values were adjusted for sex, age and BMI.

There was a significant difference in allele frequency and genotype distribution between the patients and control subjects at rs4074134. Logistic analysis with A(MA) allele as a dependent variable showed that rs4074134 was associated with T2DM irrespective of age, gender or BMI (OR = 0.87; 9%CI: 0.77–0.99; P = 0.04) (also see Supplementary Table 1).

A similar association was also observed for rs4074134 when pre-diabetes was analyzed as the case group, (OR = 0.87; 95%CI: 0.77–0.97; P = 0.01). This was also independent of age, gender and BMI. However, we found no association between T2DM or pre-diabetes and any of the other four SNP loci. Logistic regression analysis, adjusting for age, gender and BMI and combining T2DM and pre-diabetes also resulted in a significant association between rs4074134 and T2DM or pre-diabetes (OR = 0.87; 95%CI: 0.79–0.97; P = 0.009) .

### Association study on obesity

Subgroup analysis was performed to evaluate the association between SNPs (excluding rs10938397 which was not in Hardy-Weinberg equilibrium) and obesity in Chinese Han population. To remove the influence of anti-diabetic drug use on this analysis, only subjects with pre-diabetes and control subjects were included in this analysis. Based on diagnostic criteria for obesity specific for Chinese patients [19], the subjects were classified into three groups: a normal weight group (BMI<24 kg/m^2^), an overweight group (BMI 24 to 28 kg/m^2^) and an obese group (BMI≥28 kg/m^2^).

The allele and genotype distributions (shown in Supplementary Table 2) were in accordance with Hardy-Weinberg equilibrium in the normal weight group. A case control study between the normal weight and obesity group identified an association between obesity and rs4074134 in subjects with a normal OGTT (OR = 0.70; 95%CI: 0.55–0.90; P = 0.004) and the in the pooled population without T2DM (OR = 0.79; 95%CI: 0.68–0.92; P = 0.002). We also observed a non-significant association between obesity and rs17782313 in the controls subjects (OR = 1.28; 95%CI: 0.97–1.69; P = 0.08) and in the pooled group of controls and subjects with pre-diabetes (OR = 1.16; 95%CI: 0.98–1.38; P = 0.08). No association was observed between obesity and any of the other SNPs either with or without adjustment for age and gender(Table 3).

**Table 3:** Logistic regression analysis of association of five SNPs with obesity in subjects without diabetes.

SNPs		Control			Pre-diabetes			Control and Pre-diabetes		
		Case/controlMAF	OR (95% CI)	P-value	Case/controlMAF	OR(95%CI)	P-value	Case/controlMAF	OR (95% CI)	P-value
Rs2815752*	G	0.09/0.11	0.84(0.55–1.27)	0.410	0.08/0.09	0.93(0.65–1.37)	0.70	0.08/0.10	0.85(0.64–1.12)	0.25
Rs4074134	A	0.45/0.37	0.70(0.55–0.90)	0.004	0.42/0.38	0.84(0.69–1.02)	0.08	0.43/0.38	0.79(0.68–0.92)	0.002
Rs17782313	G	0.28/0.23	1.28(0.97–1.69)	0.078	0.24/0.24	1.11(0.88–1.39)	0.38	0.25/0.23	1.16(0.98–1.38)	0.08
Rs11084753	G	0.33/0.34	0.67(0.37–1.23)	0.197	0.35/0.32	1.17(0.95–1.43)	0.14	0.34/0.33	1.08(0.92–1.26)	0.35

The additive model for minor allele was used and odds ratios were calculated by logistic regression analysis.

SNP: single nucleotide polymorphism; MAF: minor allele frequency; OR: odds ratios.

P values were adjusted for sex and age.

* : The dominant model for minor allele was used.

### Genotype–phenotype association analysis

To avoid the influence of anti-diabetic treatment, genotype–phenotype association analysis was only undertaken in the control and pre-diabetes groups. In this analysis we investigated differences among genotypes of T2DM-related quantitative traits including BMI, waist circumference, fasting and postprandial plasma glucose, fasting and postprandial serum insulin, HOMA-β and HOMA-IR.

Subjects in the control group without the A allele for rs4074134 had a higher BMI than the subjects with the A allele (AA: 25.0±3.1 kg/m^2^; AG: 25.1±3.1 kg/m^2^; GG: 25.6±3.4 kg/m^2^; P = 0.029). No significant differences in other metabolic traits were found among genotypes. In the subjects with pre-diabetes, there were no significant differences among genotypes for any of the above metabolic traits (data not shown).

Analysis pooling data from control and subjects with pre-diabetes showed that BMI, waist circumference, fasting and postprandial plasma glucose, fasting serum insulin, and HOMA-IR were all higher in subjects without the A allele than in those with the A allele (Table 4).

**Table 4:** Comparison of T2DM-related traits among genotypes of rs4074134 in subjects without T2DM.

	GGN = 932	AGN = 1320	AAN = 471	*P*-value
M/F	334/598	488/832	175/296	0.830
Age, years	52±10	53±10	52±10	0.690
BMI, kg/m^2^	26.1±3.2	25.7±3.1	25.7±3.2	0.020
Waist circumference, cm	88±10	87±10	87±10	0.010
Male	92±8	91±9	91±9	0.040
Female	85±10	84±9	84±9	0.060
Fasting plasma glucose, mmol/L	5.7±0.7	5.6±0.7	5.6±0.6	0.030
2-h plasma glucose, mmol/L	7.7±2.0	7.5±2.0	7.4±2.0	0.010
Fasting serum insulin, mU/L†	8.8 (5.8–12.3)	7.9 (5.5–11.5)	8.1(5.7–12.1)	0.040
2-h serum insulin, mU/L†	33.9 (21.3–66.3)	35.6 (20.9–56.9)	30.5(20.6–47.8)	0.230
HOMA-IR†	2.2 (1.4–3.3)	1.9 (1.3–2.9)	2.0(1.3–3.0)	0.005
HOMA-β†	81.8 (57.5–117.2)	79.4 (55.2–111.3)	82.9 (56.6–121.1)	0.480

Data are shown as means ± SD or median (interquartile range). *P* represents the significance of differences among GG, AG and AA genotypes.

The distribution of males/females was analyzed using Pearson's χ^2^ test. Quantitative variables were compared using ANOVA.

BMI: body mass index; HOMA-IR: homeostasis model assessment of insulin resistance; HOMA-β: homeostasis model assessment of β cell function;

† Natural logarithm-transformed to normal distributions before statistical analysis.

Linear regression analyses adjusting for age and gender identified and associations between rs4074134 and BMI, waist circumference, fasting and postprandial plasma glucose, fasting serum insulin, and HOMA-IR. Analysis with adjustment of BMI showed that the association remained only for fasting and postprandial plasma glucose (Table 5).

**Table 5:** Linear regression analyses between rs4074134 and T2DM-related phenotypes.

Phenotype	Controls		Pre-diabetes		Control and Pre-diabetes	
	β	P	β	P	β	P
BMI	−0.077	0.01	−0.02	0.42	−0.049	0.01
Waist circumference	−0.067	0.02	−0.032	0.17	−0.055	0.002
Fasting plasma glucose	0.019	0.52	−0.038	0.12	−0.043	0.02
2-h plasma glucose (mmol/L)	0.002	0.94	−0.016	0.52	−0.044	0.02
Fasting serum insulin†	−0.008	0.80	−0.007	0.76	−0.018	0.32
Adjusting age and gender*	−0.033	0.30	−0.017	0.49	−0.038	0.054
HOMA-IR†	−0.006	0.85	−0.012	0.61	−0.025	0.16
Adjusting age and gender*	−0.031	0.33	−0.021	0.41	−0.045	0.02
HOMA-β†	−0.014	0.65	0.014	0.56	0.011	0.56
Adjusting age and gender*	−0.034	0.29	0.005	0.84	−0.005	0.80

The associations between rs4074134 and quantitative T2DM-related traits were analyzed by multiple linear regressions.

β and p values were adjusted for sex, age and/or BMI. For BMI and waist circumference, linear analyses were adjusted for sex and age.

For fasting plasma glucose, serum insulin, HOMA-IR and HOMA-β, linear analyses were adjusted for sex, age and BMI.

* adjusted for age and gender.

† For non-normally distributed variables, all the variables were natural logarithm-transformed to normal distributions before statistical analysis.

## Discussion

We evaluated the contribution of five variants near five genes of *NEGR1*, *GNPDA2*, *BDNF*, *MC4R* and *KCTD15* to the development of T2DM in a Chinese Han population. We replicated a previously reported association between *BDNF*-rs4074134 and obesity. We also observed that this SNP was associated with not only T2DM and hyperglycemia but also the other metabolic features such as waist circumference, fasting serum insulin level and insulin resistance.

Obesity is a complex condition caused by genetic and environmental factors, and is an important predicator for T2DM. *FTO* was the first susceptibility gene for obesity to be identified and together with BMI was reported to be associated with T2DM [12],[13] in European Caucasian subjects. *FTO* genetic variants have been reported to be associated with T2DM in several Asian studies. However, its role in the Asian population as a whole is not so important as in Caucasians because the risk allele frequencies are much lower in Asian than in Caucasian populations [14]–[16].

In a previous study we demonstrated an association between the *FTO* gene and T2DM in Chinese Han patients even after adjusting for BMI [15]. This observation was confirmed by a recently published large-scale meta-analysis of *FTO* in Asian subjects [17]. This hypothesis has also been verified by recent studies showing that *GNPDA2*, *BCDIN3D/FAIM2*, *SH2B1*, *FTO*, *KCTD15* and *BDNF* were associated with T2DM and BMI in Han Chinese patients in Hong Kong [20]. Other workers have shown *SEC16B, TMEM18, GNPDA2, BDNF, MTCH2, BCDIN3D–FAIM2, SH2B1–ATP2A1, FTO, MC4R, KCTD15* to be associated with T2DM and BMI in Japanese subjects [21]. In Japanese subjects, but not in Han Chinese subject in Hong Kong, *FTO, TMEM18, GNPDA2, BDNF, BCDIN3D* were associated with T2DM independently of BMI [20],[21].

Differences in environmental risk profiles, body composition, and genetic background between Caucasian and Asian subjects may explain these various findings. Asian subject have been shown to be at risk for T2DM at lower levels of obesity than Caucasians, partly due to increased predisposition to visceral adiposity [22] and reduced pancreatic β-cell function [23]. Unaccounted for differences in the treatment of T2DM also explain the inconsistent results, as insulin increases and metformin decreases BMI. Statistical adjustment for BMI may therefore have an unpredictable effect on the power to identify genetic loci of T2DM and obesity. Indeed, a recent analysis stratified according to BMI reported a novel locus in *LAMA1* for T2DM in lean subjects [24].

Most subjects with pre-diabetes eventually develop diabetes unless intervention is undertaken. In our study, we included patients with pre-diabetes as a case group, in order to reduce the interference of diabetes treatment and properly evaluate the contribution of the genetic variations associated with BMI in the pathogenesis of T2DM. We showed that *BDNF* genetic variation was associated with pre-diabetes independently of BMI, and in accordance with its association with T2DM and hyperglycemia. Subsequent phenotype and genotype relationship analysis indicated rs4074134 or other variations in linkage equilibrium might affect insulin sensitivity rather than beta cell function, which in turn may alter the risk for pre-diabetes and T2DM.

A large study in 18014 middle aged Danish subjects examined 15 genetic variations from 14 loci and showed that the A allele of rs4923461 displayed a borderline BMI-dependent protective effect on T2DM, whereas *SH2B1* rs7498665 was associated with a nominally BMI-independent increased risk of T2DM [25]. Another large-scale European study in 34840 patients with T2DM and 114981 controls identified a SNP (rs12970134) of *MC4R*, which is in strong LD with variants associated with BMI [8],[11], as a new T2DM susceptibility loci with genome-wide significance [26]. These findings suggest that different pathophysiologic pathways involved in obesity play a role in development of T2DM in different ethnic groups.

*BDNF* is a member of the neurothrophin family of growth factors [27], which has a high affinity for the tropomyosin-related kinase B (*TrkB*) receptor [28]. BDNF is abundantly expressed throughout the developing and mature CNS and in many peripheral tissues, including muscle, liver and adipose, where it modulates energy metabolism and feeding behavior [29],[30],[31], [32]. Animals with reduced *BDNF* expression due to a conditional knockout in the brain develop hyperphagia, obesity and resistance to insulin [33],[34]. Intracerebroventricular administration of *BDNF* has been shown to decrease energy intake and body weight in rats, and to reverse the hyperphagic and obese phenotype of *BDNF* mutant mice [33]. It has also been shown to lower blood glucose and enhance energy expenditure in db/db mice via activation of the sympathetic nervous system [35].

WAGR syndrome in humans is characterized by heterozygous gene deletions near *BDNF*. All affected subjects who are heterozygous for *BDNF* become obese by the age of 10 [36]. A published case report describes a de novo chromosomal inversion, 46,XX,inv(11)(p13p15.3) (a region encompassing the *BDNF* gene) in an 8-year-old girl with hyperphagia, severe obesity, impaired cognitive function, hyperactivity and low serum concentration of *BDNF* protein [37]. These findings indicate that BDNF may play an important role in energy equilibrium, which is a key pathogenic factor in obesity and T2DM.

In previous studies, five SNPs including rs4074134, rs6265 (val66met), rs4923461, rs12291063 and rs925946 [9], [11], [20], [21] have been reported to be associated with obesity or/and BMI in Caucasian and Asian subjects. Another study reported an association between homozygosity for the minor C allele at rs12291063, reduced VMN *BDNF* expression and high BMI [38]. Low circulating levels of *BDNF* have been observed in individuals with both obesity and T2DM [39]. The same workers reported that plasma levels of BDNF were decreased in humans with T2DM, independently of obesity, suggesting that BDNF may regulate obesity and insulin resistance via different mechanisms [39].

The *BDNF* gene produces transcriptions with either short or long 3′ untranslated regions (3′-UTRs). It has been shown that long 3′UTR *BDNF* mRNA was enriched in the dentrites of hypothalamic neurons and that insulin and leptin could stimulate its translation in dentrites [40]. In these studies, mice harboring a truncated long BDNF 3′UTR developed severe hyperphagic obesity. These findings provide evidence indicating a relationship between *BDNF* mRNA with a long 3′UTR, leptin, neuronal activation and body weight, suggesting targeted *BDNF* mRNA is essential for energy balance and response to leptin [40]. These variations in BDNF structure might affect its protein activity and mRNA transcription pattern in a way that increases the risk for obesity and T2DM. Chinese subjects with the risk allele of BDNF might have limited ability to resist excess food intake and subsequently develop T2DM. These subjects also have increased predisposition to visceral adiposity and reduced pancreatic β-cell function [1],[22],[23].

A meta-analysis of associations between BMI and approximately 2.4 million SNPs in 27,715 East Asian subjects with replication studies in 37,691 and 17,642 additional East Asian subjects confirmed that loci at *MC4R* and *BDNF* were associated with obesity [41]. The loci in the *GNPDA2* nearly reached the genome-wide significance [41]. Our study population was much smaller than that in the meta-analysis. However, we confirmed the association between *BDNF*, obesity and BMI but found no significant associations for the other four genetic loci. There was, however, a tendency towards an association between *MC4R*, obesity and BMI.

It is likely that our study lacked power to adequately evaluate the other four loci possibly due to differences in genetic background among races. In the published studies in Chinese Han subjects, only one study from Hong Kong [20] evaluated the relationship between these five genetic loci and T2DM, and only *ETV5/DGKG* (rs7647305) was found to be associated with T2DM independently of BMI. It is notable that the SNPs near BDNF that were studied were different from those in our investigation, also suggesting possible differences between Han populations from Hong Kong and Beijing in China.

In conclusion, we demonstrated that a common variation of *BDNF* is associated with T2DM independently of obesity in the Chinese Han population. This variant has an effect on plasma glucose concentration, BMI and insulin resistance.

## Supporting Information

Table S1 **The distribution of genotypes of five studied SNPs in subjects with normal glucose tolerance and pre-diabetes.**
(DOCX)

Table S2 **The distribution of genotypes of five studied SNPs in subjects with different body mass index.**
(DOCX)
