# Supplementary material for: Rs4074134 Near BDNF Gene Is Associated with Type 2 Diabetes Mellitus in Chinese Han Population Independently of Body Mass Index
Source: PLoS One. 2013 Feb 19;8(2):e56898. doi: 10.1371/journal.pone.0056898 (PMC3576386; doi:10.1371/journal.pone.0056898)
Supplement: Table S1 — The distribution of genotypes of five studied SNPs in subjects with normal glucose tolerance and pre-diabetes. (DOCX) [file pone.0056898.s001.docx]

Supplementary Table 1 The distribution of genotypes of five studied SNPs in subjects with normal glucose tolerance and pre-diabetes

|  | **Control** | **Type 2 diabetes** | | | | **Pre-diabetes** | | | | | | **Type 2 diabetes and Pre-diabetes** | | | | | |
| --- | --- | --- | --- | --- | --- | --- | --- | --- | --- | --- | --- | --- | --- | --- | --- | --- | --- |
| **SNPs** | **aa/Aa/AA** | **aa/Aa/AA** | **OR(95%CI)** | **P** | | **aa/Aa/AA** | | **OR(95%CI)** | | **P** | | **aa/Aa/AA** | | **OR(95%CI)** | | **P** | |
| rs2815752* | 8/197/905 | 8/202/961 | 0.61(0.21-1.76) | 0.36 | 7/268/1336 | | 0.93(0.76-1.13) | | 0.45 | | 15/470/2297 | | 0.94(0.78-1.13) | | 0.49 | |  |
| rs10938397 | 93/498/490 | 128/479/556 | 1.02(0.89-1.17) | 0.75 | 159/633/776 | | 0.94(0.83-1.66) | | 0.28 | | 287/1112/1332 | | 0.96(0.86-1.07) | | 0.47 | |  |
| rs4074134 | 208/547/344 | 193/563/409 | 0.87(0.77-0.99) | 0.03 | 263/773/588 | | 0.85(0.76-0.95) | | 0.005 | | 456/1336/997 | | 0.86(0.78-1.95) | | 0.004 | |  |
| rs17782313 | 73/388/638 | 63/409/694 | 0.94(0.82-1.09) | 0.43 | 94/574/921 | | 0.99(0.87-1.12) | | 0.83 | | 157/983/1615 | | 0.98(0.87-1.10) | | 0.67 | |  |
| rs11084753 | 128/502/463 | 157/547/459 | 1.11(0.97-1.26) | 0.12 | 197/669/719 | | 0.99(0.99-1.08) | | 0.08 | | 354/1216/1178 | | 1.01(0.91-1.12) | | 0.86 | |  |

a: minor allele; A: major allele;

SNP: single nucleotide polymorphism; MAF: minor allele frequency; OR: odds ratios. P values were adjusted for sex and age.

The additive model for minor allele was used and odds ratios were calculated by logistic regression analysis except for rs2815752.

*: The dominant model for minor allele was used
