# Supplementary material for: Rs4074134 Near BDNF Gene Is Associated with Type 2 Diabetes Mellitus in Chinese Han Population Independently of Body Mass Index
Source: PLoS One. 2013 Feb 19;8(2):e56898. doi: 10.1371/journal.pone.0056898 (PMC3576386; doi:10.1371/journal.pone.0056898)
Supplement: Table S2 — The distribution of genotypes of five studied SNPs in subjects with different body mass index. (DOCX) [file pone.0056898.s002.docx]

Supplementary Table 2 The distribution of genotypes of five studied SNPs in subjects with different body mass index

| **SNPs** | **Controls** | | | **Pre-diabetes** | | | **Controls and pre-diabetes** | | |
| --- | --- | --- | --- | --- | --- | --- | --- | --- | --- |
|  | **BMI<24** | **24≤BMI<28** | **≥28** | **BMI<24** | **24≤BMI<28** | **≥28** | **BMI<24** | **24≤BMI≤28** | **≥28** |
|  | **aa/Aa/AA** | **aa/Aa/AA** | **aa/Aa/AA** | **aa/Aa/AA** | **aa/Aa/AA** | **aa/Aa/AA** | **aa/Aa/AA** | **aa/Aa/AA** | **aa/Aa/AA** |
| rs2815752 | 3/78/313 | 3/86/423 | 2/33/167 | 3/69/351 | 3/134/625 | 2/65/363 | 6/147/664 | 6/220/1048 | 4/98/530 |
| rs4074134 | 76/197/115 | 103/260/145 | 29/89/83 | 75/208/143 | 121/370/275 | 67/198/170 | 151/405/258 | 224/630/420 | 96/287/253 |
| rs17782313 | 26/126/238 | 34/176/297 | 12/86/102 | 22/146/251 | 42/280/425 | 29/151/247 | 48/272/489 | 76/456/722 | 41/237/349 |
| rs11084753 | 43/172/170 | 69/226/209 | 16/103/83 | 40/188/189 | 93/315/336 | 63/169/194 | 83/360/359 | 162/541/545 | 79/272/277 |

a: minor allele; A: major allele; BMI: body mass index;
